# The concept of personal drugs in the undergraduate pharmacology practical curriculum

**DOI:** 10.4103/0253-7613.42308

**Published:** 2008-06

**Authors:** Gurudas Khilnani

**Affiliations:** JLN Medical College, Ajmer - 305 001, Rajasthan, India. E-mail: drgurudas@rediffmail.com

I have read the thought provoking research letter by Dr Parmar and Jadav on The concept of personal drugs in the undergraduate pharmacology practical curriculum.[1] I have the following comments to share with the readership of the IJP.

The concept of P-drug for the undergraduate practical curriculum is a good instrument to promote the practice of rational therapeutics with the objective to promote use of cost effective, safe and suitable medicines. However, the concept needs to be utilized in its right perspective so that it does not leave enough to question its utility.

In selecting a P-drug for acute amoebic dysentery so far as the drug group is concerned, nitroimidazoles may be the choice. However, choosing a drug among this group will require utilization of certain criteria. Therefore, using the criteria of efficacy, safety, suitability and affordability [Table 1], one may conclude that tinidazole is a better choice to be a P-drug (along with a luminal amoebicide-vide infra). This is because of its suitability i.e., once or twice daily intake) and comparable cost of a course of treatment (3 days for tinidazole v/s 7-10 days of metronidazole). The authors have ignored the suitability factor. It is one of the important criteria for selecting a P-drug. It not only includes patient-related conditions, which preclude the use of selected drug, but also the convenience of dosage form and dosage schedule.[2] Adherence to treatment is improved by creating a good doctor-patient relationship, giving necessary drug related information to the patient and most importantly, prescribing a few drugs with a simple dosage schedule (one or two times a day).[2]

**Table 1:** Comparison of nitroimidazoles in acute amoebic dysentery[4]

*Drug*	*Dose and duration*	*Average cost (Rs.) per course*	*Efficacy/suitability*
Metronidazole	400-800 mg 3 times a ady for 7-10 days	27-00	Equal/suitable
Tinidazole	1 G/day for 3 days	21-25	Equal/more suitable
Secnidazole	2 G single dose	35-00	Equal/suitable but costly

It is difficult to agree with the authors' proposition of ignoring the advice of clinical teachers in selecting P-drug. Medical teachers have gained expertise from long standing practice and medical students learn greatly from these experts. In fact they may provide vital information to the students about the use of existing drugs and this will help them a lot in selecting their own P-drugs. Furthermore, authors' recommendation of use of a luminal amoebicide AFTER a course of nitroimidazole is contrary to the text book recommendation of its concurrent use.[4,5]

Is a P-drug concept different from the principle of choosing a ‘drug’ of choice? The drug of choice for roundworm infestation is selected by using same criteria [Table 2].

**Table 2:** Criteria of selection of a suitable antihelminthic agent[3–4]

*Drug*	*Efficacy*	*Safety*	*Suitability*	*Cost (Rs)*
Albendazole	400 mg single dose. Repeat after 3 days in heavy infections	Generally safe; may cause nausea abdominal distress, headache, lassitude.	Oral route treats coexisting trichura, pin- worms, hook worms tablet, liquid	8-12/tablet (chewable) maximum cost
	Cure rate->90%	Not safe in pregnancy		20-24 per course
Pyrentel pamoate	11 mg/kg-usually 1.0 G single dose	Mild abdominal distress, drowsiness, headache, insomnia, safety in pregnancy unknown	Oral single dose Tab=250 mg Liquid available	18-20 per course
	Cure rate-85-100%			
Mebendazole	100 mg twice a day for 3 days	Generally safe mild GIT upset, allergy	Oral tab or liquid 100 mg tablet. Treats trichura, hookworm, pinworms also Tablet-500 mg liquid available	7-16 per course
	Cure rate-100%	Not safe in pregnancy		
Piperazine hexahydrate	75 mg/kg usually 3.5-4.0 G /day for 2 days	Generally mild headache, dizziness, neurotoxicity		8.00-10.0 per course
	Cure rate->90%	Considered safe in pregnancy		

It is clear that albendazole is a drug of choice because of convenience of single dose, comparable efficacy, safety and cost. It can also be considered to be a P-drug for roundworm infestation.
